# Supplementary material for: Distinct gut microbiome patterns associate with consensus molecular subtypes of colorectal cancer
Source: Sci Rep. 2017 Sep 14;7:11590. doi: 10.1038/s41598-017-11237-6 (PMC5599497; doi:10.1038/s41598-017-11237-6)
Supplement: Supplementary file 3 — Supplementary Table S3 [file 41598_2017_11237_MOESM3_ESM.pdf]

Title: Distinct gut microbiome patterns associate with consensus molecular subtypes of colorectal cancer

Authors: Rachel V Purcell[1], Martina Visnovska[2], Patrick J Biggs[3], Sebastian Schmeier[2], Frank A Frizelle[1]

**Gene**	**Sequence 5' → 3'**
PGT[1]	
forward primer	ATCCCCAAAGCACCTGGTTT
reverse primer	AGAGGCCAAGATAGTCCTGGTAA
B. coagulans	
forward primer	GGATTGCCCGGTTTCCTTTT
reverse primer	GGCAGGAATTGAAAGGCACA
P. gingivalis	
forward primer	GGAAGAGAAGACCGTAGCACAAGGA
reverse primer	GAGTAGGCGAAACGTCCATCAGGTC
Selenomonas Sp.16S rRNA	
forward primer	AGCTATCACCTGTCGATGGG
reverse primer	CCCGTCAACAGAGCTTTACG
F. nucleatum[1]	
forward primer	CAACCATTACTTTAACTCTACCATGTTCA
reverse primer	GTTGACTTTACAGAAGGAGATTATGTAAAAATC
P. micra 16S rRNA	
forward primer	GACGATTAATACCGCATGAGACC
reverse primer	GGCTTCCTCCTATGATACCGTC
P. stomatis 16S rRNA	
forward primer	GCGAGGGTTTGCTCAGTATTG
reverse primer	TGATATATCTGCGATGCCGC

Supplementary Table S3. Primer sequences used in qPCR analysis of target bacteria. (1) Flanagan, et al. Fusobacterium nucleatum associates with stages of colorectal neoplasia development, colorectal cancer and disease outcome. *Eur J Clin Microbiol Infect Dis* **2014,** *33* (8), 1381-90.
